# Covalently Cross-Linked Arabinoxylans Films for *Debaryomyces hansenii* Entrapment

**DOI:** 10.3390/molecules200611373

**Published:** 2015-06-19

**Authors:** Ramsés González-Estrada, Montserrat Calderón-Santoyo, Elizabeth Carvajal-Millan, Felipe de Jesús Ascencio Valle, Juan Arturo Ragazzo-Sánchez, Francisco Brown-Bojorquez, Agustín Rascón-Chu

**Affiliations:** 1 Instituto Tecnológico de Tepic, Av. Tecnológico 2595, Tepic, Nayarit 63175, Mexico; E-Mails: rglezestrada@gmail.com (R.G.-E.); montserratcalder@gmail.com (M.C.-S.); arturoragazzo@hotmail.com (J.A.R.-S.); 2 Centro de Investigación en Alimentación y Desarrollo, A.C. Carretera a La Victoria Km. 0.6, Hermosillo, Sonora 83304, Mexico; E-Mail: arascon@ciad.mx; 3 Centro de Investigaciones Biológicas del Noroeste, Km. 1 Carretera a San Juan de La Costa “El Comitán”, La Paz, BCS 23097, Mexico; E-Mail: ascencio@cibnor.mx; 4 Departamento de Polímeros, Universidad de Sonora, Blvd. Luis Encinas y Rosales S/N, Col. Centro, Hermosillo, Sonora 83000, Mexico; E-Mail: fbrown@guaymas.uson.mx

**Keywords:** arabinoxylans, rheology, microscopy, cells protection

## Abstract

In the present study, wheat water extractable arabinoxylans (WEAX) were isolated and characterized, and their capability to form covalently cross-linked films in presence of *Debaryomyces hansenii* was evaluated. WEAX presented an arabinose to xylose ratio of 0.60, a ferulic acid and diferulic acid content of 2.1 and 0.04 µg∙mg^−1^ WEAX, respectively and a Fourier Transform Infra-Red (FT-IR) spectrum typical of WEAX. The intrinsic viscosity and viscosimetric molecular weight values for WEAX were 3.6 dL∙g^−1^ and 440 kDa, respectively. The gelation of WEAX (1% *w*/*v*) with and without *D. hansenii* (1 × 10^7^ CFU∙cm^−2^) was rheologically investigated by small amplitude oscillatory shear. The entrapment of *D. hansenii* decreased gel elasticity from 1.4 to 0.3 Pa, probably by affecting the physical interactions between WEAX chains. Covalently cross-linked WEAX films containing *D. hansenii* were prepared by casting. Scanning electron microscopy images show that WEAX films containing *D. hansenii* were porous and consisted of granular-like and fibre microstructures. Average tensile strength, elongation at break and Young’s modulus values dropped when *D. hansenii* was present in the film. Covalently cross-lined WEAX containing *D. hansenii* could be a suitable as a functional entrapping film.

## 1. Introduction

Arabinoxylans are important non-starch cereal polysaccharides constituted by a linear backbone of β-(1→4)-linked D-xylopyranosyl units to which α-l-arabinofuranosyl substituents are attached through *O*-2 and/or *O*-3 [1]. Some of the arabinose residues are ester linked on (*O*)-5 by ferulic acid (FA, 4 hydroxy-3-methoxycinnamic acid) [2]. These polysaccharides are classified as water extractable (WEAX) or water-unextractable (WUAX). WEAX form highly viscous solutions and can gellify through covalent ferulic acid cross-linking upon oxidation by some chemical or enzymatic free-radical-generating agents [3,4]. Laccase (*p*-diphenol oxygen oxidoreductase, EC 1.10.3.2), a blue multi-copper enzyme of white rot fungi [5] oxidizes FA from WEAX resulting in the formation of five different diFA structures (5-5ʹ-, 8-5ʹ benzo-, 8-*O*-4ʹ-, 8-5ʹ- and 8-8ʹ di-FA), the 8-5 and the 8-*O*-4 forms being always preponderant [6,7,8]. The presence of a trimer FA structure (tri-FA) in WEAX gels has been also reported [9]. This covalent WEAX cross-linking has commonly been considered as responsible for the WEAX network development, even if weak interactions also contribute to the final gel properties [10,11]. WEAX gels present interesting functional properties such as stability to temperature and pH changes, as well as neutral taste and odor, which are all desirable properties for industrial applications. As dietary fibers, WEAX and WUAX present potential health benefits for lipid metabolism, colon function, and reduction of heart disease, among others [9,10]. In addition, arabinoxylans’ ability to form a continuous and cohesive matrix has led to their use as film formers [12]. 

In recent years there has been a growing consumer interest in health, nutrition, food safety and environmental issues. This has led to renewed interest in food packaging based on natural macromolecules has been due to concerns about the environment, a decrease in fossil resources, and a need to reduce the amount of disposable packaging materials. All this has led companies and researchers to explore alternatives such as polysaccharide-based film applications in food systems [13]. Nevertheless, one of the major problems in polysaccharide films applications is their stability during thermal processing. Most currently used polysaccharide films are stabilized by physical (hydrogen bonding and/or ionic) interactions, while films based on polysaccharide covalent networks such as gelled cross-linked arabinoxylans are not common. 

Polysaccharide films can be used to protect perishable food products from deterioration [14]. It has been reported that antimicrobial compounds or biocontrol microorganisms can be incorporated into films to maintain the stability of food storage [15]. Incorporation of biocontrol microorganisms into the films could improve the control of postharvest diseases. In this regard, the capacity of the yeast *Debaryomyces hansenii* to control blue mold decay of lemon has been demonstrated [16]. Nevertheless, no research has been reported on the incorporation of microorganisms into oxidatively cross-linked WEAX films. The objective of this research was to investigate the incorporation of *D.* *hansenii* in laccase-induced WEAX films and to evaluate the morphological and mechanical properties of the material formed. 

## 2. Results and Discussion

### 2.1. Extraction and Characterization of WEAX

Yield of WEAX extracted from wheat flour was 0.45% (*w*/*w*) on a dry matter basis (db, w WEAX/w wheat flour). Similar WEAX yield values have been reported for flours of different wheat varieties [17,18]. In the present study, wheat flour WEAX were used as a convenient model, but more complex commercial arabinoxylans will be tested later on in order to make these films viable for foodstuffs as they must have a competitive price.

WEAX composition is presented in Table 1. The arabinoxylans content of the extract was estimated from the sum of xylose + arabinose as 65% db, which is close to the value reported for other wheat WEAX [18]. A residual amount of glucose was also quantified. The FA content (2.1 μg∙mg^−1^ WEAX) was in the range reported for other wheat WEAX [3,4,8]. Small levels of di-FA have been detected in WEAX (0.04 μg∙mg^−1^ WEAX), suggesting that some arabinoxylan chains might be cross-linked as previously reported [19,20,21]. The percentages of each one of the different di-FA presents in the WEAX were 80, 16, and 4% for the 8-5ʹ (mainly in the benzofuran form), 8-*O*-4ʹ, and 5-5ʹ structures, respectively. The 8-8ʹ dehydrodimer was not detected in this study. The predominance of 8-5ʹ and 8-*O*-4ʹ di-FA structures has also been reported in arabinoxylans from wheat and barley flour [6,7,8]. The tri-FA 4-*O*-8ʹ, 5ʹ-5ʺ was detected only in trace amounts. The degree of substitution (arabinose to xylose ratio, 0.60) was characteristic of wheat endosperm arabinoxylans (0.53–0.7) [9,10]. The intrinsic viscosity ([η]) and viscosimetric molecular weight (Mv) values were 3.6 dL∙mg^−1^ and 440 kDa, respectively, which are in the range previously reported for other wheat WEAX [4].

**Table 1:** Composition of water extractable arabinoxylans.

Component	Content
Arabinose ^a^	25.00 ± 1.50
Xylose ^a^	41.00 ± 1.10
Glucose ^a^	3.90 ± 0.30
Protein ^a^	3.70 ± 0.05
Ferulic acid ^b^	2.100 ± 0.001
Diferulic acids ^b^	0.040 ± 0.001
Triferulic acid ^b^	traces

^a^ Results are expressed in g/100 g WEAX dry matter; ^b^ Phenolics are expressed in g∙mg^−1^ WEAX dry matter; All results are obtained from triplicate runs.

**Figure 1:**
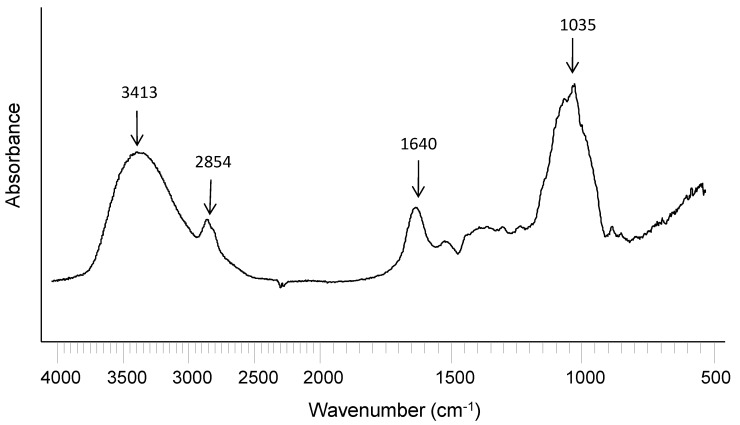
FT-IR spectrum of non-cross-linked WEAX.

The Fourier transform infrared (FTIR) spectrum of WEAX (Figure 1) shows absorbance bands for polysaccharides at 1200–800 cm^−1^, a main band centered at 1035 cm^−1^ that could be assigned to C-OH bending, with small shoulders at 1158, and 897 cm^−1^ that have been related to the antisymmetric C-O-C stretching mode of the glycosidic link and β(1→4) linkages [22]. The amide I band (1640 cm^−1^), related to protein content, which is mainly assigned to polypeptide carbonyl stretching, was also detected [23]. The band at 3413 cm^−1^ corresponds to stretching of the OH groups and the one at 2854 cm^−1 ^corresponds to the CH_2_ groups [24]. 

**Figure 2:**
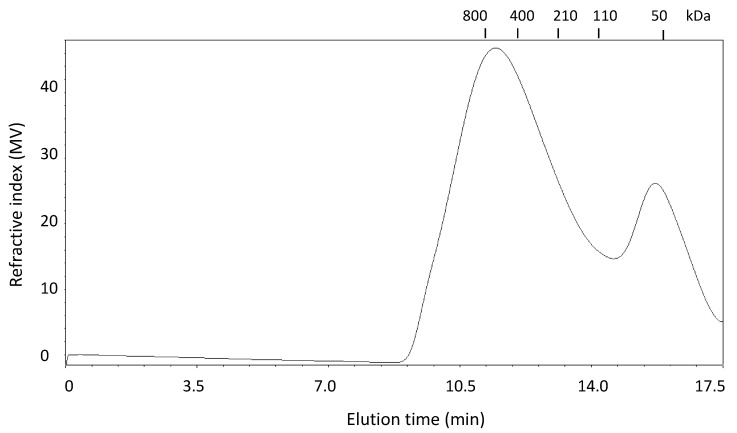
Elution profiles of WEAX. Pullulan molecular weight markers (kDa) used as calibration scale are shown at the top.

The gel permeation chromatography profile of WEAX is presented in Figure 2. The molecular weight distribution profile shows a major peak in the high molecular weight region (retention time between 10 and 14 min). A shoulder was registered to the right of the major peak indicating the presence of a WEAX population with low molecular weight values. This molecular weight distribution pattern has been previously reported for wheat WEAX [17].

### 2.2. WEAX Gelation

**Figure 3:**
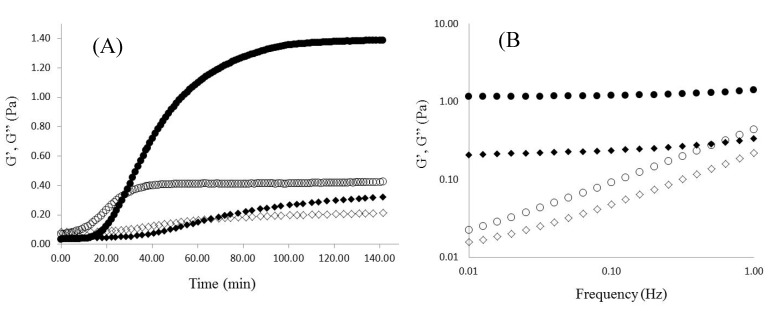
(**A**) Rheological kinetics of 1% (*w*/*v*) WEAX solution gelation by laccase, measurements at 25 °C, 1 Hz and 10% strain; (**B**) Mechanical spectrum of WEAX gel, measurements at 25 °C, 1 Hz and 10% strain. WEAX G’ (○), G” (●) and WEAX containing yeast G’ (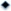), G” (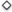).

**Figure 4:**
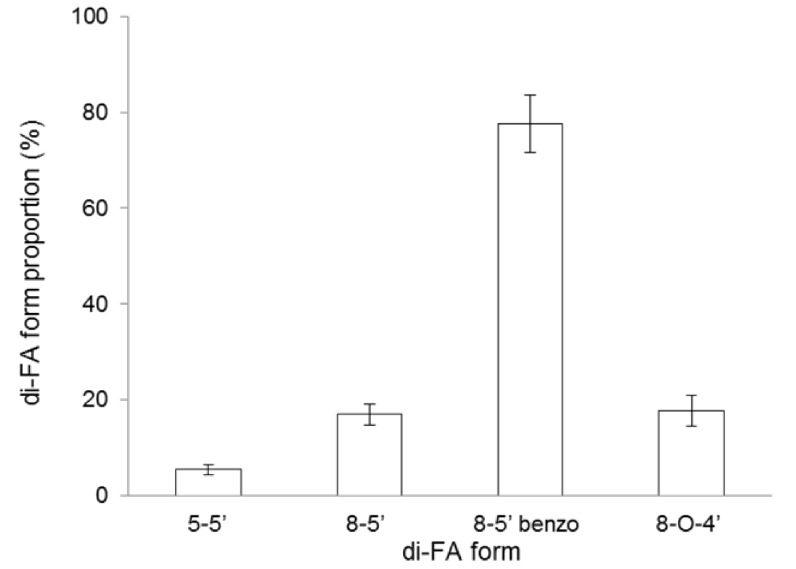
Di-FA form and proportion in WEAX gels.

The cross-linking process of WEAX was rheologically investigated by small amplitude oscillatory shear. Figure 3A shows the development of G’ and G” moduli *vs.* time of 1% (*w*/*v*) WEAX solution undergoing oxidative gelation by laccase. Storage (G’) and loss (G”) moduli rise to reach a pseudoplateau region. The final G’ and G” values of 1% (*w*/*v*) for gels were 1.4 and 0.4 Pa, and for gels containing yeast 0.3 and 0.2 Pa, respectively. This decrease in G’ could be related to loss, or weakening in WEAX chains physical interactions which may reduce the network connectivity, as previously suggested in WEAX gels containing *Bifidobacterium longum* [25]. Nevertheless, yeast cells and bacteria are rather different (in surface properties, size, shapes, *etc*.), which may lead to different interactions with the WEAX network. The gelation time (tg), calculated from the crossover of the G’ and G” curves (G’ > G”) was 31 min and for gels containing yeast 69 min. The tg value indicates the sol/gel transition point and at this point G’ = G” or tan δ = G”/G’ = 1 [26]. The mechanical spectra of WEAX after 3 h gelation (Figure 3B) was typical of solid-like materials with a linear G’ independent of frequency and G” much smaller than G’ and dependent of frequency [27]. This behavior is similar to that previously reported for arabinoxylans gels cross-linked by laccase or peroxidase/H_2_O_2_ system [10]. During WEAX gelation, ferulic acid was oxidized leading to the formation of covalent cross-linking structures (0.122 µg of di-FA per milligram of WEAX and traces of tri-FA). The 8-5ʹ benzofuran form, 8-5ʹ, 8-*O*-4ʹ and 5-5ʹ dimers represented 66%, 15%, 14% and 5% of the total di-FA amounts respectively (Figure 4). The main increase in di-FA concerned the 8-5ʹ benzofuran form. The predominance of 8-5ʹ benzofuran form and 8-5ʹ dimers and absence of the 8-8ʹ structure was also observed in other WEAX gels [7,9,11]. The amounts of di-FA and tri-FA produced did not counterbalance the lost in FA. Therefore, at the end of gelation, 63% of the initial FA in the WEAX solution disappeared, with only 37% recovered as di-FA. Low ferulate recovery after oxidative treatment of arabinoxylans has been previously reported [7,9,11]. This behavior could be explained by the possible formation of other ferulate structures which are not determined by the method used in the present study.

### 2.3. WEAX Films

**Figure 5:**
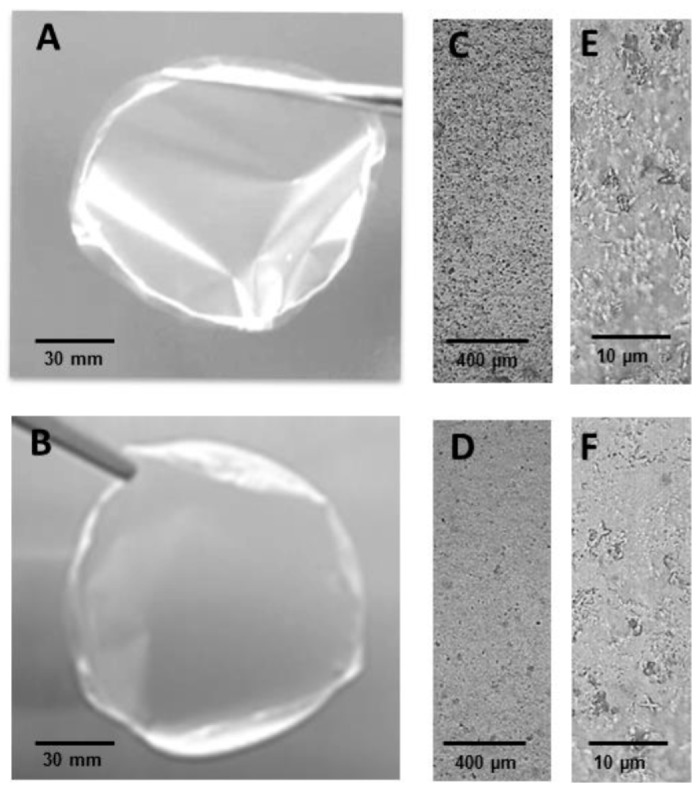
WEAX film (**A**) WEAX film entrapping *D. hansenii*; (**B**) optical micrographs of WEAX film (**C**,**E**) and WEAX film entrapping *D. hansenii* (**D**,**F**) at 4× (**C**,**D**) and 40× (**E**,**F**) magnification.

WEAX gelation system with or without *D. hansenii* formed films when was left to dry on Petri dishes. The films were slightly yellow, but still transparent, flexible, homogeneous, and with smooth surfaces (no pores or cracks). The thickness of WEAX films was 25.7 ± 1.27 and 24.1 ± 3.25 µm for WEAX films containing yeast. Figure 5A,C,E shows WEAX film while images 5(B,D,F) correspond to WEAX film entrapping *D. hansenii*. Figure 5C–F show stereomicrographs of the WEAX films surface, which are without apparent fractures. Without any oxidative gelation of the WEAX, the material formed presents a discontinuous surface and became far too fragile to handle. 

Film color can be an important factor in terms of consumer acceptance of both edible and inedible films. The L, a and b color values of WEAX film and WEAX/*D. hansenii* film are shown in Table 2. Significant differences (*P* < 0.05) in L, a, b values were detected among the films. The main difference in color values among these materials was the increased b value (yellowish) in presence of yeast cells. Color changes due to incorporation of *D. hansenii* can be more fully described using other color function such as YI which indicates degree of yellowness [28]. The entrapment of *D. hansenii* in WEAX film resulted in a significantly increase (*P* < 0.05) in YI. 

**Table 2:** Color values (L, a, and b), and yellowness index (YI) of WEAX film and WEAX film containing *D. hansenii*.

	WEAX Film	WEAX Film Containing *D. hansenii*
L	95.86 ± 0.89a	94.63 ± 0.06b
a	–0.15 ± 0.03a	–0.27 ± 0.04b
b	4.62 ± 0.27a	9.77 ± 0.16b
YI	6.88 ± 0.44a	14.75 ± 0.25b

All results are obtained from triplicates.

**Figure 6:**
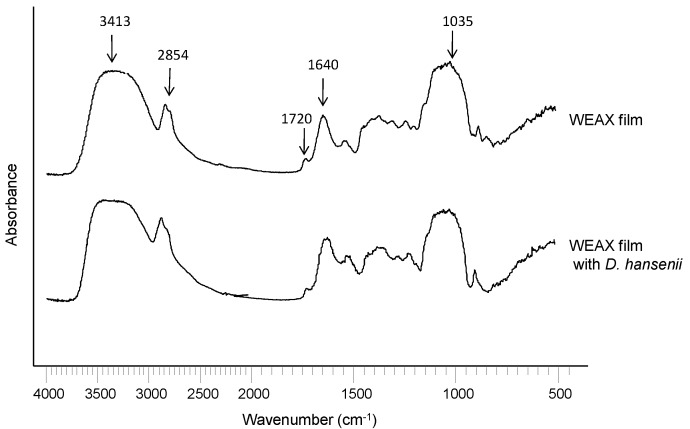
FT-IR spectra of cross-linked WEAX film and cross-linked WEAX film containing *D. hansenii*.

In the present study, FT-IR spectra of WEAX film and WEAX film containing yeast cells are shown in Figure 6. In general, film spectra were similar to those of non-cross-linked WEAX (Figure 1). However, in cross-linked WEAX a small absorption band at 1720 cm^−1^ was observed, which represents the carbonyl stretching vibrations of esters [23,24]. It has been reported that the intensity of the absorption band at 1414 cm^−1^ for CO asymmetric stretching increases after WEAX cross-linking in lyophilized microspheres [29]. However, no significant increase in this band was found in WEAX films, which could be attributed to the lower amount of polysaccharide used for the sample preparation (WEAX solutions at 1%, *w*/*v*) in comparison to that used for WEAX microspheres (WEAX solution at 4%, *w*/*v*). The FTIR spectrum of WEAX film containing *D. hansenii* was also investigated because the protein from yeast cell could favor the formation of protein-WEAX adducts which have been reported and seen by FTIR [30]. Nevertheless, these adducts were not observed in WEAX film containing yeast cell, which could be explained by the relatively low amount of entrapped cells.

**Figure 7:**
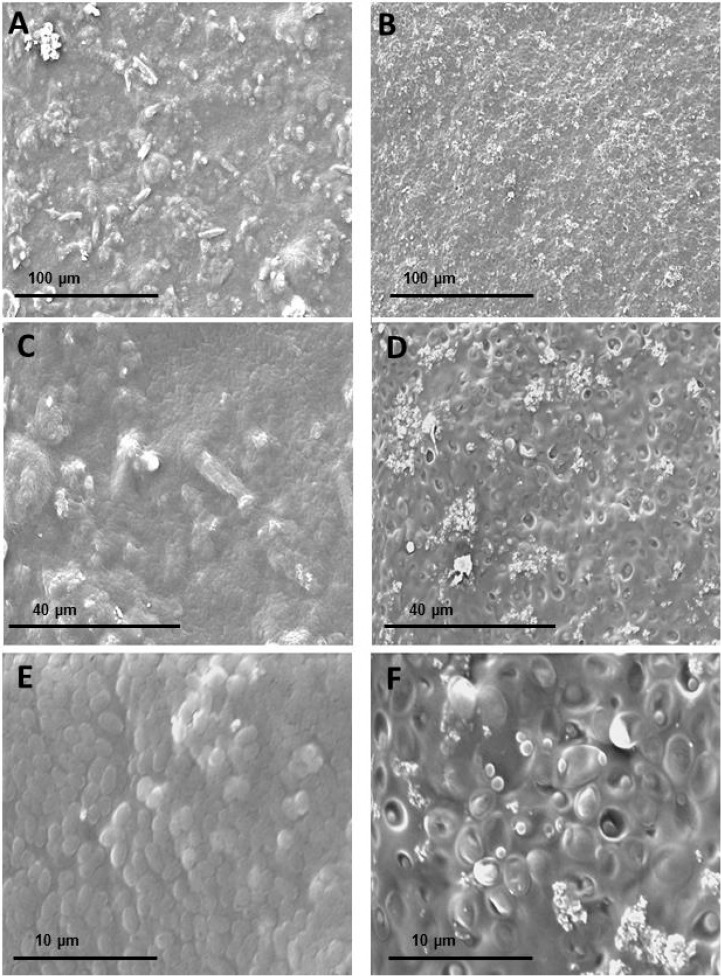
SEM micrographs of WEAX films (**A**,**C**,**E**) and WEAX films containing *D. hansenii* (**B**,**D**,**F**) at 500× magnification (A,B), 1500× magnification (**C**,**D**) and 3500× magnification (**E**,**F**).

SEM images of the WEAX film containing *D. hansenii* at different scales are presented in Figure 7. The surface morphology reflects the complexity of interactions within the WEAX film. The film surface presents some unevenness on the microscopic level which could be attributed to the high viscosity of WEAX solution in the final stages of gel formation and/or to the presence of yeast cells suspended in the WEAX gelation system. It is possible to observe the presence of granular-like and fibre microstructures that may be attributed to WEAX chain aggregates. A higher magnification clearly shows that the WEAX film contains pseudo-circular structures of 1–3 µm size, which can be attributed to entrapped yeast cells. SEM images show that WEAX films containing *D. hansenii* are porous. This morphological microstructure is similar to the microstructure of films made from corn hull arabinoxylans [31], xylans [32] and glucans [33]. From a general point of view, the porosity of WEAX films could be a relevant parameter because this characteristic may prolong *D. hansenii* viability and thus improve the inhibitory efficiency of the films on the control of postharvest diseases. 

Average tensile strength, elongation at break and Young’s modulus values dropped when *D. hansenii* was present in the WEAX film, although it should be noted that the standard deviation for the test samples was high (Table 3). It is possible that the presence of *D. hansenii* could act as defects in the WEAX films and contribute to the early breaking of the samples during tensile testing. These findings are in good agreement with the decrease in G’ of the WEAX gels entrapping yeast (Figure 3).

WEAX and WEAX containing *D. hansenii* mechanical properties were in the range reported for non-oxidative coupled arabinoxylan films, however such films were prepared by using polysaccharide solutions at higher concentrations (7.5% *w*/*v*) [31] while in the present study a WEAX solution at 1% (*w*/*v*) was used. Another study reported non oxidative coupled arabinoxylan films presenting higher tensile strength values but lower elongation at break (4.7%) than the films formed in the present study [34]. It has been reported that the polysaccharide covalent cross-linking improves the mechanical properties of the films formed [35]. Our results suggest that oxidative coupling of WEAX enhance the mechanical properties of the films formed.

**Table 3:** Mechanical properties of WEAX films.

Film	Tensile Strength (MPa)	Elongation at Break (%)	Young’s Modulus (MPa)
WEAX film	7.00 ± 1.00	12.80 ± 2.0	410 ± 51
WEAX film with *D. hansenii*	6.00 ± 1.70	10.30 ± 1.0	375 ± 10

All results are obtained from triplicate experiments.

## 3. Experimental Section 

### 3.1. Materials

Water extractable arabinoxylans (WEAX) were extracted from wheat flour which was kindly provided by a wheat milling industry in Northern Mexico (Molino La Fama). *D. hansenii* was obtained from seawater samples collected at a depth of 100 m at the Cortez Sea (Baja California, Mexico) and belonging to the Yeast Collection of the CIBNOR S.C. Commercial laccase (benzenediol:oxygen oxidoreductase, E.C.1.10.3.2) was from *Trametes versicolor*. All chemical reagents were purchased from Sigma Chemical Co. (St Louis, MO, USA). 

### 3.2. WEAX Extraction and Characterization 

WEAX were extracted as described previously [17]. Laccase activity was measured at 25 °C from a laccase solution at 0.125 mg∙mL^−1^ dissolved in 0.05 M citrate-phosphate buffer pH 5.5 as previously reported [9]. Neutral sugar content in WEAX was determined by hydrolysis of the polysaccharides with 2 N trifluoroacetic acid at 120 °C for 2 h as reported before [17]. Samples were filtered through 0.2 µm (Whatman) and analysed by HPLC using a Supelcogel Pb column (300 × 7.8 mm; Supelco, Inc., Bellefonte, PA, USA) eluted with 5 mM H_2_SO_4_ (filtered 0.2 µm, Whatman) at 0.6 mL∙min^−1^ and 50 °C. A Varian 9012 HPLC (Varian, St. Helens, Australia) equipped with q Varian 9040 refractive index detector and a Star Chromatography Workstation system control version 5.50 were used.

Ferulic acid (FA), dimers of ferulic acid (di-FA) and trimers of ferulic acid (tri-FA) contents were determined in WEAX and WEAX gel after saponification by RP-HPLC [9,11]. An Alltima C18 column (250 × 4.6 mm) (Alltech Associates, Inc., Deerfield, IL, USA) and a photodiode array detector Waters 996 (Millipore Co., Milford, MA, USA) were used. Detection was followed by UV absorbance at 320 nm.

The protein content in the WEAX powder was determined according to the Dumas method [36], using a Leco-FP 528 nitrogen analyzer (Leco, St. Joseph, MI, USA).

Viscosity measurements were made by determination of the flow times of WEAX solutions in water (from 0.06 to 0.1% *w*/*v*). An Ubbelohde capillary viscometer at 25 ± 0.1 °С immersed in a temperature controlled water bath was used. The intrinsic viscosity ([η]) was estimated from relative viscosity measurements (η rel) of WEAX solutions by extrapolation of Kraemer and Mead and Fouss curves to “zero” concentration [9]. The viscosimetric molecular weight (Mν) was calculated from the Mark-Houwink relationship, Mν = ([η]/k)1/α.

Molecular weight distribution of WEAX was determined by Size Exclusion-High Performance Liquid Chromatography (SE-HPLC) at 38 °C using a TSKgel (Polymer Laboratories, Shropshire, UK) G500 PMWX column (7.8 × 300 mm). A Water 2414 refractive index detector was used for detection. Isocratic elution was performed at 0.6 mL∙min^−1^ with 0.1 M LiNO_3_ filtered through 0.2 μm [17].

FT-IR spectra of dry WEAX and WEAX film powder were recorded on a Nicolet FT-IR spectrophotometer (Nicolet Instrument Corp., Madison, WI, USA). The samples were pressed into KBr pellets (2 mg sample/200 mg KBr). A blank KBr disk was used as background. Spectra were recorded between 400 and 4000 cm^−1^ [22].

### 3.3. WEAX Gelation

A WEAX solution (1% *w*/*v*) was prepared in distilled water pH 5. Laccase (1.675 nkat per mg WEAX) was added to WEAX solution as cross-linking agent. Gels were allowed to develop for 3 h at 25 °C [9]. WEAX solution concentration and laccase amount used were based on previous reports [4,9,11]. The advantage of using laccase as cross-link agent is that this enzyme is generally recognized as safe (GRAS Notice 000122).

Small amplitude oscillatory shear was used to follow the gelation process of WEAX solution. Cold (4 °C) WEAX solution (1% *w*/*v*) in distilled water pH 5 was mixed with laccase and immediately poured on plate-plate geometry (4.0 cm in diameter) of a strain controlled rheometer (Discovery Hybrid Rheometer, TA Instruments, New Castle, DE, USA). Exposed edges were recovered with silicone to prevent evaporation. WEAX gelation was started by a sudden increase of temperature from 4 to 25 °C and monitored at 25 °C for 2 h by recording the storage (G’) and loss (G”) moduli. Measurements were carried out at 1.0 Hz frequency and 10% strain. From strain sweep tests, WEAX gels showed a linear behavior from 0.02 to 100% strain. 10% strain was used in all the rheological measurements. The mechanical spectra of gels were obtained by frequency sweep from 0.01 to 10.0 Hz with a 10% strain at 25 °C. The same conditions were applied to WEAX solution containing *D. hansenii* [25].

### 3.4. WEAX Films Preparation and Characterization

A WEAX solution (1% *w*/*v*) and a WEAX solution (1% *w*/*v*) containing *D. hansenii* (1 × 10^8^ CFU∙mL^−1^) were prepared in distilled water (pH 5) as reported previously [25]. Laccase (1.675 nkat per mg WEAX) was used as cross-linking agent. The amount of yeast cells used was based on a previous report about WEAX gels containing *Bifidobacterium longum* [25]. Glycerol (0.05% w/w WEAX) was used as plasticizer. Dispersions were poured over polyethylene Petri dishes (90 mm in diameter) covered with a 1.5 mm thickness Teflon film to facilitate the recovering of the dried films. The dispersions were left to dry approximately 36 h at room temperature and low relative humidity in a desiccator. WEAX films formed presented a thickness value of 0.02 ± 0.001 mm, measured with a micrometer (Code No. 293–230, Mitutoyo Corp., Kawasaki, Kanagawa, Japan). WEAX film color was evaluated with a colorimeter (Minolta CR400, Ramsey, NJ, USA) and standardized with respect to white calibration plate. Colorimeter provided CIE L, a, and b values. L is lightness, and a (−greenness to +redness) and b (−blueness to +yellowness) are the chromaticity coordinates. Yellowness index (YI) was calculated [28]. Four readings were taken at different locations on each film. Measurements were done in triplicate. FT-IR spectra of WEAX films were recorded on a Nicolet FT-IR spectrophotometer (Nicolet Instrument Corp.). The films were placed in a sample holder. Air (blank) was used as background. Spectra were recorded between 400 and 4000 cm^−1^. WEAX films were frozen at −20 °C and lyophilized at −37 °C/0.133 mbar overnight in a Freezone 6 freeze drier (Labconco, Kansas, MO, USA). The structure of the WEAX films was analyzed with a Motic BA300Pol microscope (Motic Incorporation Ltd., Hong Kong, China) at low magnifications (4× and 40×). The structure of freeze-dried WEAX films was studied by scanning electron microscopy (JEOL 5410LV, Peabody, MA, USA) at low voltage (20 kV). SEM image was obtained in secondary electrons image mode. Tensile tests were carried out using a TA-XT2 texture analyser (Stable Micro Systems, Godalming, England). Film strips (14 mm × 50 mm) were attached on tensile grips and stretched at 0.5 mm∙s^−1^ in tension mode [37]. 

## 4. Conclusions 

Oxidative cross-linked WEAX film was achieved for the first time. In addition, in the present study, it was possible to entrap *D. hansenii* in the WEAX film. The presence of yeast results in a slight reduction of WEAX gel elasticity, probably by affecting the physical interactions taking place between WEAX chains. Average tensile strength, elongation at break and Young’s modulus values drop when *D. hansenii* was present in the WEAX film. WEAX films are porous and consist of granular-like and fibre microstructures. The results suggest that oxidative cross-linked WEAX films can be potential candidates for the entrapment of yeast as a functional entrapping film. WEAX films barrier properties, water stability and yeast viability will be investigated during the second part of the project, where the WEAX film entrapping *D. hansenii* will be tested as a biological control of blue mold decay in Mexican lemon. 

## References

[1] Dervilly-Pinel G., Thibault J.F., Saulnier L. (2001). Experimental evidence for a semi-flexible conformation for arabinoxylans. Carbohydr. Res..

[2] Smith M.M., Hartley R.D. (1983). Occurrence and nature of ferulic acid substitution of cell-wall polysaccharides in graminaceous plants. Carbohydr. Res..

[3] Izydorczyk M.S., Biliaderis C.G., Bushuk W. (1991). Physical properties of water-soluble pentosans from different wheat varieties. Cereal Chem..

[4] Figueroa-Espinoza M.C., Rouau X. (1998). Oxidative cross-linking of pentosans by a fungal laccase and a horseradish peroxidase: Mechanism of linkage between feruloylated arabinoxylans. Cereal Chem..

[5] Bollag J.M., Leonowicz A. (1984). Comparative studies of extracellular fungal laccase. Appl. Environ. Microb..

[6] Ralph J., Quideau S., Grabber J.H., Hatfield R.D. (1994). Identification and synthesis of new ferulic acid dehydrodimers present in grass cell walls. J. Chem. Soc. Perkin Trans. 1.

[7] Figueroa-Espinoza M.C., Morel M.H., Rouau X. (1998). Effect of lysine, tyrosine, cysteine and glutathione on the oxidative cross-linking of feruloylated arabinoxylans by a fungal laccase. J. Agric. Food Chem..

[8] Schooneveld-Bergmans M.E.F., Dignum M.J.W., Grabber J.H., Beldman G., Voragen A.G.J. (1999). Studies on oxidative cross-linking of feruloylated arabinoxylans from wheat flour and wheat bran. Carbohydr. Polym..

[9] Carvajal-Millan E., Guigliarelli B., Belle V., Rouau X., Micard V. (2005). Storage stability of arabinoxylan gels. Carbohydr. Polym..

[10] Izydorczyk M.S., Biliaderis C.G. (1995). Cereal arabinoxylans: advances in structure and physicochemical properties. Carbohydr. Polym..

[11] Vansteenkiste E., Babot C., Rouau X., Micard V. (2004). Oxidative gelation of feruloylated arabinoxylan as affected by protein. Influence on protein enzymatic hydrolysis. Food Hydrocoll..

[12] Chanliaud E., Saulnier L., Thibault J.F. (1995). Alkaline extraction and characterization of heteroxylans from maize bran. J. Cereal Sci..

[13] Pérez Espitia P.J., Du W.X., Avena-Bustillos R.J., Ferreira Soares N.F., McHugh T.H. (2014). Edible films from pectin: Physical-mechanical and antimicrobial properties—A review. Food Hydrocoll..

[14] Biliaderis C.G., Gerasopoulos D., Sfakiotakis E. (2001). Physicochemical properties and application of pullulan edible films and coatings in fruit preservation. J. Sci. Food Agric..

[15] Fan Y., Xu Y., Wang D., Zhang L., Sun J., Sun L., Zhang B. (2009). Effect of alginate coating combined with yeast antagonist on strawberry (Fragaria × ananassa) preservation quality. Postharvest Biol. Technol..

[16] Hernández-Montiel L., Larralde-Corona C., Vero S., López-Aburto M., Ochoa J., Ascencio-Valle F. (2010). Characterization of yeast *Debaryomyces hansenii* for the biological control of blue mold decay of Mexican lemon. CyTA-J. Food.

[17] Carvajal-Millan E., Landillon V., Morel M.H., Rouau X., Doublier J.L., Micard V. (2005). Arabinoxylan gels: impact of the feruloylation degree on their structure and properties. Biomacromolecules.

[18] Izydorczyk M., Biliaderis C.G., Bushuk W. (1991). Comparison of the structure and composition of water-soluble pentosans from different wheat varieties. Cereal Chem..

[19] Saulnier L., Sado P.E., Branlard G., Charmet G., Guillon F. (2007). Wheat arabinoxylans: exploiting variation in amount and composition to develop enhanced varieties. J. Cereal Sci..

[20] Dervilly G., Saulnier L., Roger P., Thibault J.F. (2000). Isolation of homogeneous fractions of wheat water-soluble arabinoxylan. Influence of structure on their macromolecular characteristics. J. Agric. Food Chem..

[21] Fincher G.B., Stone B.A. (1974). A water-soluble arabinogalactan–peptide from wheat endosperm. Aust. J. Biol. Sci..

[22] Barron C., Rouau X. (2008). FTIR and Raman signatures of wheat grain peripheral tissues. Cereal Chem..

[23] Cyran M.R., Saulnier L. (2005). Cell wall fractions isolated from outer layers of rye grain by sequential treatment with α-amylase and proteinase: Structural investigation of polymers in two ryes with contrasting breadmaking quality. J. Agric. Food Chem..

[24] Séné C.F.B., McCann M.C., Wilson R.H., Grinter R. (1994). Fourier-Transform Raman and Fourier-Transform Infrared Spectroscopy. An investigation of five higher plant cell walls and their components. Plant Physiol..

[25] Morales-Ortega A., Carvajal-Millan E., Brown-Bojorquez F., Rascón-Chu A., Torres-Chavez P., López-Franco Y.L., Lizardi-Mendoza J., Martínez-López A.L., Campa-Mada A.C. (2014). Entrapment of probiotics in water extractable arabinoxylan gels: Rheological and microstructural characterization. Molecules.

[26] Doublier J.L., Cuvelier G., Eliasson A.C. (1996). Gums and hydrocolloids: Functional aspects. Carbohydrates in Food.

[27] Ross-Murphy S.B., Chan H.W.S. (1984). Rheological methods. Biophysical Methods in Food Research.

[28] Bolin H.R., Huxsoll C.C. (1991). Control of minimally processed carrot (*Daucus carota*) surface discoloration caused by abrasion peeling. J. Food Sci..

[29] Martínez-López A.L., Carvajal-Millan E., López-Franco Y.L., Lizardi-Mendoza J., Rascón-Chu A., Haghi A.K., Carvajal-Millan E. (2014). Antioxidant activity of maize bran arabinoxylan microspheres. Food Composition and Analysis. Methods and Strategies.

[30] Boeriu C.G., Oudgenoeg G., Spekking W.T., Berendsen L.B., Vancon L., Boumans H., Gruppen H., van Berkel W.J., Laane C., Voragen A.G. (2004). Horseradish peroxidase-catalyzed cross-linking of feruloylated arabinoxylans with beta-casein. J Agric. Food Chem..

[31] Zhang P., Whistler R.L. (2004). Mechanical properties and water vapor permeability of thin film from corn hull arabinoxylan. J. Appl. Polym. Sci..

[32] Goksu E.I., Karamanlioglu M., Bakir U., Yilmaz L., Yilmazer U. (2007). Production and characterization of films from cotton stalk xylan. J. Agric. Food Chem..

[33] Novák M., Synytsya A., Gedeon O., Slepička P., Procházka V., Synytsya A., Blahovec J., Hejlová A., Čopíková J. (2012). Yeast β(1,3),(1,6)-d-glucan films: Preparation and characterization of some structural and physical properties. Carbohydr. Polym..

[34] Rouilly A., Geneau-Sbartaï C., Rigal L. (2009). Thermo-mechanical processing of sugar beet pulp. III. Study of extruded films improvement with various plasticizers and cross-linkers. Bioresour. Technol..

[35] Höije A., Sternemalm E., Heikkinen S., Tenkanen M., Gatenholm P. (2008). Material properties of films from enzymatically tailored arabinoxylans. Biomacromolecules.

[36] AOAC (1995). Official Methods of Analysis of AOAC Intl. Method 991.43.

[37] Alves V., Ferreira A., Costa N., Freitas F., Reis M., Coelhoso I. (2011). Characterization of biodegradable films from the extracellular polysaccharide produced by *Pseudomonas oleovorans* grown on glycerol byproduct. Carbohydr. Polym..

